# Arthroscopy vs. MRI for a detailed assessment of cartilage disease in osteoarthritis: diagnostic value of MRI in clinical practice

**DOI:** 10.1186/1471-2474-11-75

**Published:** 2010-04-20

**Authors:** Lars V von Engelhardt, Matthias Lahner, André Klussmann, Bertil Bouillon, Andreas Dàvid, Patrick Haage, Thomas K Lichtinger

**Affiliations:** 1 Department of Trauma and Orthopedic Surgery, HELIOS-Klinikum Wuppertal, Heusnerstr. 40, 42283 Wuppertal, University of Witten/Herdecke, Witten, Germany; 2 Department of Orthopedic and Trauma Surgery, St Josef Hospital, Gudrunstrasse 56, Ruhr-University Bochum, 44791 Bochum, Germany; 3 Institute of Occupational Health, Safety and Ergonomics (ASER) at the University of Wuppertal, Corneliusstrasse 31, 42329 Wuppertal, Germany; 4 Department of Trauma and Orthopedic Surgery, Medical Center Cologne-Merheim, Ostmerheimerstr. 200, 51109 Cologne, University of Witten/Herdecke, Witten, Germany; 5 Department of Diagnostic and Interventional Radiology, HELIOS-Klinikum Wuppertal, Heusnerstr. 40, 42283 Wuppertal, University of Witten/Herdecke, 42283 Wuppertal, Germany

## Abstract

**Background:**

In patients with osteoarthritis, a detailed assessment of degenerative cartilage disease is important to recommend adequate treatment. Using a representative sample of patients, this study investigated whether MRI is reliable for a detailed cartilage assessment in patients with osteoarthritis of the knee.

**Methods:**

In a cross sectional-study as a part of a retrospective case-control study, 36 patients (mean age 53.1 years) with clinically relevant osteoarthritis received standardized MRI (sag. T1-TSE, cor. STIR-TSE, trans. fat-suppressed PD-TSE, sag. fat-suppressed PD-TSE, Siemens Magnetom Avanto syngo MR B 15) on a 1.5 Tesla unit. Within a maximum of three months later, arthroscopic grading of the articular surfaces was performed. MRI grading by two blinded observers was compared to arthroscopic findings. Diagnostic values as well as intra- and inter-observer values were assessed.

**Results:**

Inter-observer agreement between readers 1 and 2 was good (kappa = 0.65) within all compartments. Intra-observer agreement comparing MRI grading to arthroscopic grading showed moderate to good values for readers 1 and 2 (kappa = 0.50 and 0.62, respectively), the poorest being within the patellofemoral joint (kappa = 0.32 and 0.52). Sensitivities were relatively low at all grades, particularly for grade 3 cartilage lesions. A tendency to underestimate cartilage disorders on MR images was not noticed.

**Conclusions:**

According to our results, the use of MRI for precise grading of the cartilage in osteoarthritis is limited. Even if the practical benefit of MRI in pretreatment diagnostics is unequivocal, a diagnostic arthroscopy is of outstanding value when a grading of the cartilage is crucial for a definitive decision regarding therapeutic options in patients with osteoarthritis.

## Background

Osteoarthritis is the most common joint disorder, characterized by an imbalance between synthesis and degradation of the articular cartilage with destruction of the joint [1]. For patients with mild to severe diseases, increasing numbers of surgical and non-surgical treatment modalities, such as analgetic treatment, application of hyaluronic acids and growth factors, cartilage transplants, osteochondral transfers, microfracturing, corrective osteotomies or partial and total knee replacements, have gained popularity [2-5]. In regard to this wide range of therapeutic options, it has become increasingly difficult to recommend adequate treatment. In clinical practice, a detailed assessment of disease severity includes an exact grading of the cartilage. Therefore, a reliable non-invasive visualization of cartilage disorders becomes important and may be an additional support in the decision, which therapeutic options should be suggested. Several studies comparing the value of cartilage diagnostics on MRI to intra-operative findings present very different results. In a large part of the studies, diagnostic values of MRI were assessed by collapsing several grades of cartilage disorders into a disease positive and a disease negative status [6-15]. This simplification mostly does not correspond to surgeons' demands of an exact staging of cartilage disorders. Additionally, only a few comparative clinical studies have focused on degenerative cartilage diseases [16-22]. These studies, which compare MRI grading and arthroscopic grading of the cartilage in patients with knee osteoarthritis, used different statistical methods and demonstrated various results [16-22]. Blackburn et al. showed a "moderate" correlation of MRI grading and arthroscopic grading (Pearson correlation coefficient r = 0.4) [17]. In contrast, two other studies demonstrated a "highly significant" correlation using the Spearman rank correlation test (*P *> 0.0003 and *P *> 0.0001, respectively) [18,20]. When intra- and inter-observer agreements were assessed, the kappa values ranged between "slight" and "very good" agreement [17,19,21]. The diagnostic values reported in clinical studies and cadaver studies also present a wide range, with sensitivities ranging from 31% to 100% [16,20,22]. The aim of this study was to determine whether MRI is a reliable method for a detailed assessment of the articular cartilage in patients with advanced and clinically relevant osteoarthritis or not. For this purpose, MR images and intra-operative findings in a representative sample of patients were evaluated.

## Methods

### Subjects

This cross-sectional study is part of a research project where influencing factors for the onset of knee osteoarthritis were determined in a case-control study. Inclusion criteria were defined to assess a representative sample with clinically relevant and advanced knee osteoarthritis. Thus, only patients with a radiological ≥ grade 2 disease on the Kellgren and Lawrence scale on x-rays and at least grade 3 disease on the Outerbridge grading, assessed during arthroscopy, were included [23,24]. Furthermore, all patients included in this study were treated for clinically relevant and ongoing symptoms of knee osteoarthritis in our hospital. Clinical relevance was defined as having lead to any medical treatment, e.g. for pain reduction. Before being referred to our hospital, patients included in this study received conservative treatments. Because of persistent complaints being refractory to drugs, all patients included in this study required knee surgery. Patients with previous knee trauma, such as meniscal or ligamentous tears, cartilage injuries and fractures as well as patients with inflammatory or reactive knee joint diseases were excluded [25]. A study protocol was prepared and approved by the Ethics Committee of the University of Witten/Herdecke. All patients included in this study gave written informed consent. During the period from July 2006 to June 2008, 576 in-patients were treated for symptomatic knee osteoarthritis at the Helios-Klinikum Wuppertal. Of these, 436 patients fulfilled the inclusion criteria for our case-control study. Furthermore, only patients who received standardized MRI at our institution and subsequent arthroscopy within a maximum delay of three months were included in this study. Thus, the final sample size used in this study consisted of 36 patients (18 female, 18 male) with a mean age of 53.1 years.

### MR imaging

**Figure 1:**
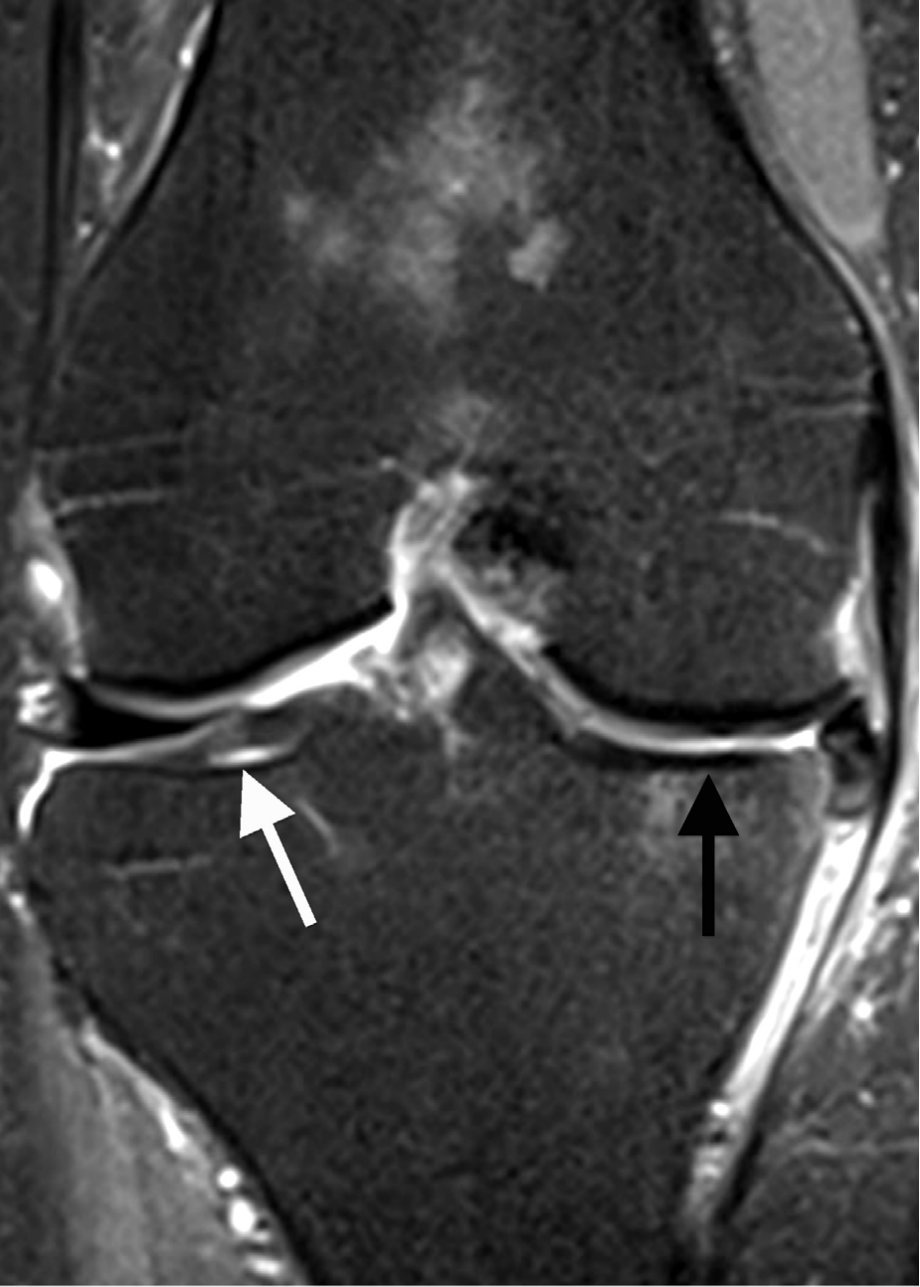
**Coronal PD-weighted TSE MRI of a 68-year-old woman**. Tibial medial MRI shows a full-thickness defect of the cartilage with denudation of the bone (black arrow). This finding is defined as a grade 4 disorder. Signal heterogeneities within the cartilage at the lateral tibia were documented as a grade 1 cartilage disease (white arrow).

**Figure 2:**
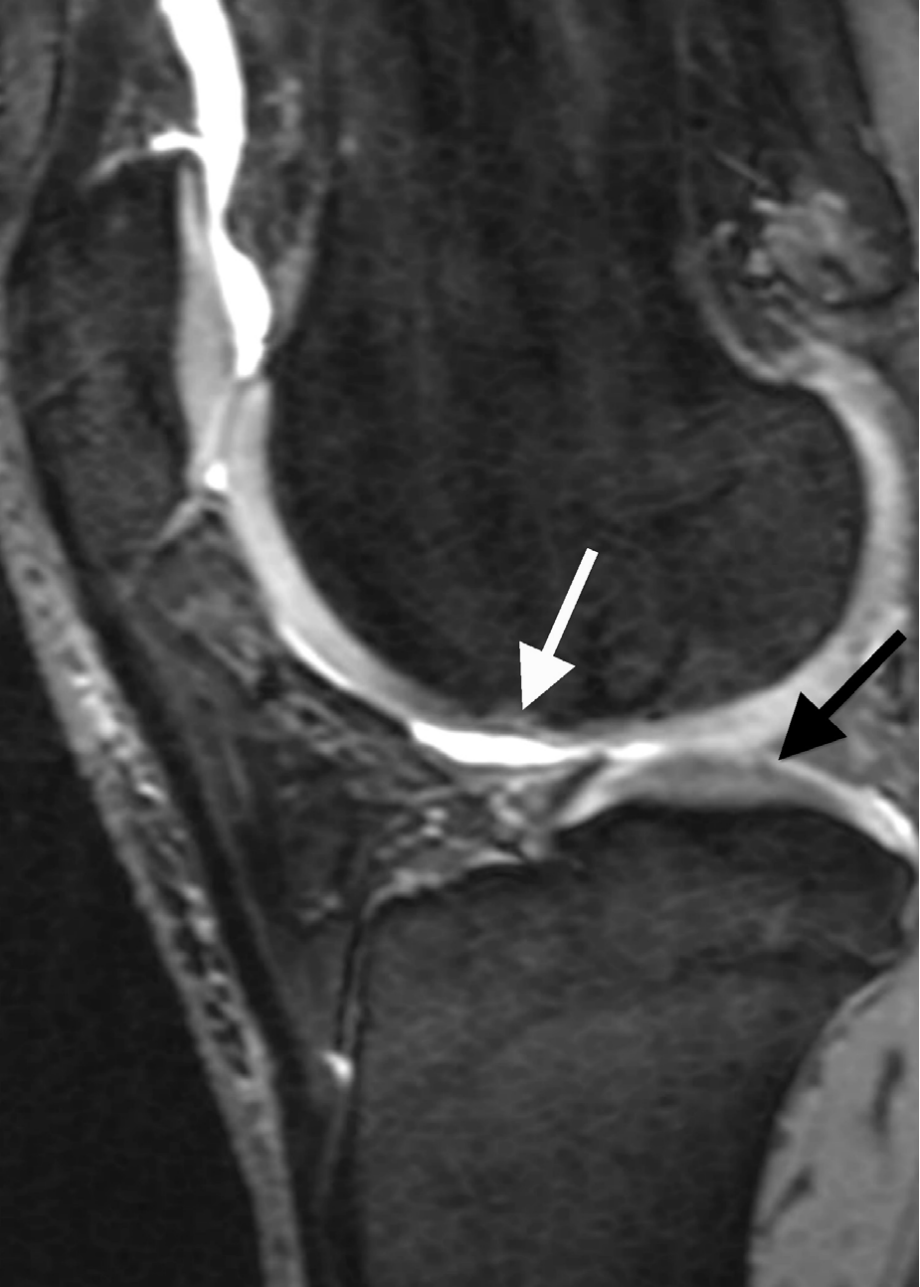
**Sagittal PD-weighted TSE MRI of a 41-year-old male**. A defect of more than 50% of the cartilage thickness at the medial femoral condyle is visible (white arrow). This finding is defined as grade 3 disease. A grade 2 disorder as a superficial fibrillation or erosion composing less than 50% of the cartilage was noticed at the tibial medial plateau (black arrow).

The average period between MRI and arthroscopy was 28.9 days (range two to 90 days). Patients underwent MR imaging at a 1.5-Tesla system (Siemens Magnetom Avanto syngo MR B 15) with a maximum gradient strength of 15 mT/m (rise time 0.2 msec, slew rate 150 mT/m/msec). A flexible synergy surface coil with two coil elements was used for imaging and was placed anteriorly and posteriorly to the knee. The following sequences were used in this study: a T1-weighted turbo spin-echo sequence (T1-TSE) in sagittal planes [field of view (FOV): 160 mm, matrix: 384, resolution: 0.4 mm × 0.4 mm × 0.6 mm, slices: 20, slice thickness: 4 mm, repetition time (TR): 461 ms, echo time (TE): 12 ms, flip angle (FA): 90°, acquisition time (AT): 4:29 min], a short tau inversion recovery sequence (STIR), TSE in coronal planes (FOV: 160 mm, matrix: 256, resolution: 0.8 mm × 0.6 mm × 4.0 mm, slices: 20, slice thickness: 4 mm, TR: 5100 ms, TE: 27 ms, FA: 160°, AT: 5:43 min), a transversal proton density (PD) weighted TSE with fat suppression (FOV: 150 mm, matrix: 256, resolution: 0.6 mm × 0.6 mm × 3.0 mm, slices: 20, slice thickness: 3 mm, TR: 965 ms, TE: 26 ms, FA: 40°, AT: 4:09 min) as well as a PD-weighted TSE with fat suppression in sagittal planes (FOV: 160 mm, matrix: 256, resolution: 0.6 mm × 0.6 mm × 4.0 mm, slices: 20, slice thickness: 4 mm, TR: 951 ms, TE: 26 ms, FA: 40°, AT: 4:05 min). MR images were reviewed separately on a PACS workstation (ID. Read, Image Devices) by two orthopedic surgeons experienced in diagnostics and treatment of knee osteoarthritis (L. v. E. and T. K. L). Both were blinded to clinical data, including surgical reports. To compare the MRI results to those found at arthroscopy, the articular surface of the knee was divided into six regions: patella, trochlea, medial femoral condyle, medial tibia, lateral femoral condyle and lateral tibia. Each cartilage surface was analyzed as a single entity. To perform a direct comparison between MRI and arthroscopy, we used a classification based on the Outerbridge macroscopic grading. This MRI classification was used in several previous studies [10,26,27]. Grade 0 is defined as cartilage with a normal intrinsic signal and a normal surface contour on MR images. Signal heterogeneities within the cartilage in the presence of a smooth surface were rated as grade 1 lesions and conform to the arthroscopic finding of a cartilage softening (Figure 1). A grade 2 disorder shows a fibrillation or erosion composing less than 50% of the cartilage thickness (Figure 2). Defects of more than 50% are defined as grade 3 and occur with or without small bone ulcerations (Figure 2). Extended full-thickness lesions with denudation of the bone are defined as grade 4 (Figure 1). In cases of multiple cartilage defects within one of the six articular surfaces, only the highest grade of cartilage damage was documented.

### Arthroscopy

All patients had a standardized 1.5-Tesla MRI examination and subsequent arthroscopic surgery after a maximum delay of three months. Arthroscopic grading of cartilage disorders was performed by six orthopedic surgeons experienced in knee surgery. At the time of arthroscopy, the MR images were available to the surgeon, whereas the MRI grading of the hyaline cartilage was not present. Surgery was performed by using the standard antero-medial and antero-lateral portals. Each knee compartment was inspected thoroughly and palpated using a blunt hook. Arthroscopic findings were classified in grades 0 to 4 according to the system of Outerbridge [24]. Cartilage lesions were recorded in a standardized documentation sheet derived from the mapping method employed by the International Cartilage Repair Society (ICRS) [28]. Cartilage damage was treated in the same session with abrasion (15 patients), microfracturing (12 patients) and drilling (2 patients) or open surgery with osteochondral transfers (2 patients) as well as unicondylar (3 patients) and bicondylar arthroplasty (2 patients).

### Statistical analyses

Sensitivity, specificity, accuracy, negative predictive values of MRI and 95% confidence intervals of the estimated values were calculated using arthroscopic findings as reference standard. Weighted kappa values for multiple categories and 95% confidence intervals were calculated to assess intra- and inter-observer agreement [29]. According to Landis and Koch, a kappa value of 0.20 or less indicates a poor agreement; 0.21-0.40, fair; 0.41-0.60, moderate; 0.61-0.80, good; and 0.81-1.0, very good agreement [30]. Statistical analyses were made using the software program Graph pad prism 3.0 (Graph pad software, La Jolla, CA).

## Results

According to the inclusion criteria, advanced cartilage disorders were predominant in our study. Of 216 joint surfaces, 85 (39%) were assessed as grade 0, 10 (5%) as grade 1, 31 (14%) as grade 2, 50 (23%) as grade 3 and 40 (19%) as grade 4 lesions during arthroscopy.

MRI gradings of both reviewers were compared to our arthroscopic findings, and weighted kappa values for multiple categories were calculated (Table 1). There was an exact agreement between arthroscopic findings and MRI readings in 123 of 216 joint surfaces (57%) for reader 1 and in 102 of 216 (47%) for reader 2. As presented in Table 1, intra-observer agreement differed in the three compartments of the knee. The poorest results were obtained in the patellofemoral compartment. In cases of disagreement, the tendency to under- or overestimate the severity of cartilage damage on MR imaging was evaluated in a further count. Compared to arthroscopic findings, cartilage diseases were undergraded by readers 1 and 2 in 23% (50 of 216) and 25% (53 of 216) and overgraded in 20% (43 of 216) and 28% (61 of 216) of cases, respectively. Gradings of readers 1 and 2 showed exact inter-observer agreement in 126 of 216 cases (58%). Inter-observer agreements between readers 1 and 2 varied markedly for the three different compartments. A second course of MR images evaluation was performed in order to assess the agreement when the observers repeated the MRI grading. For all knee compartments, kappa values and 95% confidence intervals were 0.75 (0.69-0.81) for reader 1 and 0.73 (0.67-0.79) for reader 2. Thus, a "good" agreement for duplicate MRI grading of the cartilage was noticed consistently for both readers.

**Table 1:** Weighted kappa values and 95% confidence intervals for inter- and intra-observer agreement of MRI for the three compartments of the knee joint.

	weighted kappa values and 95% confidence intervals^†^			
	**patellofemoral compartment**	**medial compartment**	**lateral compartment**	**all compartments**

reader 1 vs. reader 2	0.51 (0.37-0.65)	0.59 (0.47-0.71)	0.75 (0.64-0.85)	0.65 (0.58-0.72)
AC vs. reader 1	0.52 (0.37-0.66)	0.62 (0.48-0.75)	0.65 (0.54-0.76)	0.62 (0.55-0.69)
AC vs. reader 2	0.32 (0.17-0.47)	0.49 (0.35-0.62)	0.57 (0.44-0.70)	0.50 (0.42-0.58)

* AC = arthroscopic findings

^† ^< 0.20 = poor, 0.21-0.40 = fair, 0.41-60 = moderate, 0.61-0.80 = good, 0.81-1.0 = very good

In a further investigation, the diagnostic values of MRI grading, using arthroscopy as reference standard, were calculated for each grade of cartilage damage (Table 2). For grade 1, 2 and 3 lesions, sensitivities were relatively poor, whereas relatively better values were noted for grade 4 disorders.

**Table 2:** Both observers' diagnostic values of MRI for each grade of cartilage degeneration and 95% confidence intervals considering arthroscopy as reference standard.

	grade I	grade II	grade III	grade IV
True Positive Findings	2	16	18	28
	2	15	15	24

False Negative Findings	8	15	32	12
	8	16	35	16


False Positive Findings	10	40	19	11
	16	49	25	9

True Negative Findings	196	145	147	165
	190	136	141	167

Sensitivity [%]	20 (6-47)	52 (36-67)	36 (26-46)	70 (58-79)
	20 (6-48)	48 (33-64)	30 (20-41)	60 (48-69)

Specificity [%]	95 (95-96)	78 (76-81)	89 (86-92)	94 (91-96)
	92 (92-94)	74 (71-76)	85 (82-88)	95 (92-97)

negative predictive value [%]	96 (95-97)	91 (88-94)	82 (79-85)	93 (91-95)
	96 (95-97)	90 (86-93)	80 (77-83)	91 (89-93)

accuracy [%]	92 (90-94)	75 (70-79)	76 (72-81)	89 (85-93)
	89 (88-42)	70 (66-74)	72 (68-77)	88 (84-92)

## Discussion

In our study, the diagnostic values for MRI assessment of cartilage lesions were relatively low at all grades of disease. The assessment of inter-observer agreement between readers 1 and 2 revealed mostly moderate and good results (weighted kappa = 0.51-0.75). For the intra-observer agreement, when comparing MRI grading to arthroscopic grading, slightly poorer results with fair, moderate and good values (weighted kappa = 0.32-0.65) were demonstrated. Previous clinical studies focused on patients with osteoarthritis showed different values for the agreement of MRI grading and arthroscopic grading of cartilage damage. Drapé et al. demonstrated very good intra- and inter-observer agreements (weighted kappa = 0.91 and 0.64, respectively). Based on this data, MRI was proposed as an outcome measure of cartilage lesions in clinical trials treating osteoarthritis [19]. In contrast to this study, McNicholas et al. noted mainly "slight" or "fair" intra- and inter-observer agreements for the evaluation of cartilage damage in patients with knee osteoarthritis. In regard to this data, reservations about the use of MRI in the assessment of disease severity were stated [21]. Similarly to a study of Blackburn et al. [17], our results, lie in the mid range, demonstrating moderate to good kappa values (Table 1). There are several possibilities to explain the differences between these studies and our study. Regarding patient selection, the inclusion criteria for clinically relevant osteoarthritis were clearly defined in the study of Drapé et al. and Blackburn et al. [17,19], whereas no inclusion criteria were set in the study of McNicholas et al. [21]. Furthermore, it has to be mentioned that MRI techniques used in the studies were quite different. Drapé et al. used a 0.2-T musculoskeletal dedicated MR unit (Artoscan; Esaote Biomedica) with two successive 3D gradient-echo sequences and 1.4 mm slice thickness [19], whereas McNicholas et al. used a FISP 3 D gradient echo sequence on a 1.0 T scanner (Siemens) and a dedicated surface coil without further description of slice thickness [21]. Similarly to our study, Blackburn et al. used proton density-, T1- and T2-weighted spin-echo sequences with 4 mm slice thickness on a 1.5 T scanner and a standard extremity coil [17]. The comparable MRI techniques used in the study of Blackburn et al. and our study as well as comparable inclusion criteria are possibly the cause for similar results. A better diagnostic performance reported in the study of Drapé et al. could be explained by a relatively thin slice thickness. Furthermore, the performance reported in this study could be explained by the use of two 3D gradient-echo sequences, which were thought to be advantageous for the detection of cartilage lesions [6,11,15,31,32]. Even so, the MR sequence best suited for the detection of chondral abnormalities is still under debate [33,34]. The fact that MR images were available to the surgeon at arthroscopy could further influence our results. At arthroscopy, the MRI visualization of osteoarthritis, which is often combined with additional findings, such as osteophytes, bone marrow edema, sclerosis, cysts, etc. could affect the intra-operative grading of the cartilage, even if the MRI grading of the hyaline cartilage was not present at the time of arthroscopy. Interestingly, intra-observer agreements were poorest in the patellofemoral compartment. This could be explained by the so-called "magic angle effect", which influences the visualization of the cartilage at certain orientations of collagen fibers corresponding to the magic angle of 55° [35]. This phenomenon, often occurring at the articular poles of the patella, can influence the accurate interpretation of these areas [36]. A poor inter-observer agreement at arthroscopy could also impair the validity of our study, where several orthopedic surgeons were involved. Studies about inter-observer agreement at arthroscopy demonstrate fair and moderate inter-observer agreement, particularly for the patellofemoral cartilage [37,38], but also sufficient reproducibility [39]. In this context, it has to be mentioned that a comparable intra-operative cartilage assessment is an important objective within most orthopedic departments, especially regarding patients with osteoarthritis. Therefore, a standardized cartilage grading and mapping, as described in the methods chapter, is routinely performed within our clinic. This consistent grading of the cartilage, which has been asserted for years, possibly leads to familiar use of this classification with comparative results within our clinic. However, appropriate appraisement of these studies, which compare the grading of cartilage among surgeons of several countries, clinics, etc., seems to be difficult. Nevertheless, a study of Acebes et al., which evaluated the agreement between arthroscopic and histopathological grading, shows much better results. This study revealed that the arthroscopic method is a valuable tool in clinical research to score chondropathies in the medial femorotibial compartment in knee osteoarthritis [40].

Bachmann et al. studied degenerative cartilage changes in trauma patients with a mean age of 29 years. A significant tendency to underestimate the severity of damage on MR images was reported [16]. In further studies comparing MRI- and arthroscopic findings, degenerative cartilage disorders were undergraded more often than overgraded [17,18]. Broderick et al. concluded, that this tendency to underestimate cartilage disorders must be taken into account when clinical and research procedures are being planned [18]. According to our study protocol, only patients with clinically relevant osteoarthritis were included. This leads to a relatively high frequency of advanced cartilage disorders with numerous possibilities for underestimation. In our series, undergrading during MRI assessment was noticed for both observers in 23% and 25% of cases, respectively; and overgrading was assessed in 20% and 28% of cases, respectively. Thus, even though advanced cartilage disorders were relatively frequently compared to the studies mentioned above, this tendency could not be confirmed in our study.

Regarding the patients included in this study, a detailed assessment of degenerative cartilage disorders was important to recommend adequate treatment. For these patients, MRI was expected to be an additional decision support. In the literature, only few studies evaluate diagnostic relevance of MRI in patients with degenerative cartilage diseases. Review of these studies revealed a high variability of diagnostic values. Kawahara et al. demonstrated the best results, showing a sensitivity of 32% for grade 1, 72% for grade 2, 94% for grade 3 and 100% for grade 4 disorders [20]. MR scans were performed on a 0.5 T unit. Similarly to our study, MR images were obtained with fast spin-echo imaging. In a further own study using fast spin-echo sequences on a 3-Tesla MRI unit, sensitivity was 26% for grade 1, 63% for grade 2, 64% for grade 3 and 77% for grade 4 lesions [22]. In contrast to the present study, only patients with at least grade 2 lesions and a negative trauma anamnesis were included. Further inclusion criteria were not defined. Thus, the present study better reflects patients with clinically relevant degenerative disorders of the cartilage, where decision-making sometimes appears difficult in clinical practice. Bachmann et al. reported a sensitivity of 14% for grade 1, 32% for grade 2, 94% for grade 3 and 100% for grade 4 lesions [16]. A disadvantage of this study is that mainly athletes with a mean age of 29 years suffering from an acute knee disorder were included. Therefore, a comparison to elderly patients with clinically relevant osteoarthritis could be difficult. Our diagnostic values for the detection of grade 1 and 2 lesions are reasonably within the ranges of the aforementioned studies, whereas the values for grades 3 and 4 were relatively poor. Especially the diagnostic values for the detection of grade 3 lesions, showing a sensitivity of only 30% and 36%, were disappointing. Specificities and negative predictive values, which were relatively good for all other grades, also showed poor results for grade 3 lesions. The slice thickness of 3 and 4 mm, used in our MRI protocol, might possibly affect the ability of MRI to detect a small grade 3 lesion, whereas the visualization of a grade 2 disorder, which is frequently extended over a large articular surface, is less affected. Furthermore, the thickness of the hyaline cartilage, which was reported to measure between 2 and 3 mm [41], could complicate a correct MRI cartilage diagnosis when a relatively broad slice thickness is chosen. Interestingly, in the study of Kawahara et al., which demonstrated the best diagnostic values, MR images were obtained with a slice thickness of 5 mm. Using fast spin-echo sequences, protocols chosen in this study were similar to our study [20]. Thus, poor diagnostic values in our study could not be solely explained by the slice thickness of 3 and 4 mm. Because our sensitivities show relatively low values at all grades of cartilage disease, it has to be assumed that the application of MRI for a precise grading of degenerative cartilage disorders is limited (Table 2). As a consequence, it has to be claimed that arthroscopy should not be replaced by MRI when a grading of the cartilage is crucial for the definitive planning of a therapeutic procedure. In these cases, a diagnostic arthroscopy should be suggested even if MR images are available. This point of view could be mirrored by the treatment procedures of some patients included in this study. In our series, arthroscopy was performed in five patients before uni- or bicondylar arthroplasties were implanted during the same surgery. In these cases, we had reservations about the reliability of MRI findings even though MR images were subjectively of quite good quality.

Regarding therapeutic decisions, controversial positions on surgical proceedings should be mentioned. On the one hand, several studies demonstrate that arthroscopic treatments in knee osteoarthritis may delay more extensive surgery such as replacement arthroplasty [42,43]. On the other hand, a large evaluation of over 14.000 arthroscopic debridement procedures for knee osteoarthritis revealed that almost 10% of patients required total knee replacement within one year. Because rates of arthroplasty were high particularly in elderly patients, an overutilization of arthroscopic treatments in this patient group was discussed [44]. In this context, the unequivocal value of MRI for the visualization of further typical findings of osteoarthritis, such as osteophytes, bone marrow edema, subchondral sclerosis, cysts, etc. has to be mentioned. For instance, it has been shown that osteophytes and joint effusions detected at MRI were significantly associated to clinical features such as pain and stiffness [45-48]. Certainly, such MRI findings should be considered and adjusted to clinical investigation. Possibly, further aspects of MR imaging could support the individual decision-making regarding treatments in patients with knee osteoarthritis. With respect to the difficulties for an exact assessment of disease severity in osteoarthritis patients encountered herein, further studies on this complex topic must further query the value of MRI in clinical practice.

## Conclusions

In patients with osteoarthritis, the value of MRI for a precise grading of the cartilage is limited. When the assessment of the cartilage is crucial for a definitive decision regarding therapeutic options in patients with osteoarthritis, our data suggest that arthroscopy should not be generally replaced by MRI.

## Competing interests

The authors declare that they have no competing interests.

## Authors' contributions

LVvE, AK, AD, BB and PH conceived and designed the study. In addition, LVvE and TKL performed MRI grading; LVvE, ML, PH, AK, TKL and AD are involved in the execution of the study and the writing of this manuscript. All authors read and approved the final manuscript.

## Pre-publication history

The pre-publication history for this paper can be accessed here:

http://www.biomedcentral.com/1471-2474/11/75/prepub
